# Self-perceived oral health, general self-efficacy, and their relations to oral health conditions in head and neck cancer patients in Sweden—a prospective observational study

**DOI:** 10.3389/froh.2025.1693673

**Published:** 2026-01-05

**Authors:** Charlott Karlsson, Niklas Bohm, Caterina Finizia, Jessica Skoogh Andersson, Annica Almståhl

**Affiliations:** 1 Faculty of Odontology, Malmö University, Malmö, Sweden; 2 Department of Oral and Maxillofacial Surgery, Institute for Postgraduate Dental Education, Jönköping, Sweden; 3 Department of Oral Microbiology and Immunology, Institute of Odontology, Sahlgrenska Academy, University of Gothenburg, Gothenburg, Sweden; 4 Department of Otorhinolaryngology Head and Neck Surgery, Institute of Clinical Sciences, Sahlgrenska Academy, University of Gothenburg, Gothenburg, Sweden; 5 Department of Otorhinolaryngology, Head and Neck Surgery, Sahlgrenska University Hospital, Gothenburg, Region Västra Götaland, Sweden; 6 Department of Periodontology, Institute of Odontology, Sahlgrenska Academy, University of Gothenburg, Gothenburg, Sweden

**Keywords:** head and neck cancer, oral health promotion, oral hygiene, oral mucositis, radiotherapy, self-efficacy, self-perceived oral health

## Abstract

**Objective:**

To explore self-perceived oral health and general self-efficacy and relate this to dental plaque, gingival inflammation, and oral mucositis in head and neck cancer patients in Sweden, before, during, and three months after treatment.

**Methods:**

Registration of clinical variables (dental plaque, gingival inflammation, and oral mucositis) was performed in 75 patients. The patients completed the self-perceived oral health (SPOH), and general self-efficacy (GSE) questionnaires at baseline, week 6 during treatment, and 3 months after treatment. Changes in clinical variables and answers to questionnaires between time-points were analyzed as well as differences between clinical variables and questionnaire data.

**Results:**

The majority had low levels of plaque and gingival inflammation at all time-points, and oral mucositis occurred in 82%. Forty-three percent perceived their oral health as good at baseline, and the proportion decreased to 18% at week 6. At baseline, toothbrushing twice a day was reported by 95%, and daily interdental cleaning by 51%. The majority had high self-efficacy at all time-points. No statistically significant differences between self-perceived oral health and clinical variables were found.

**Conclusion:**

Despite major challenges during cancer treatment, most patients had good oral hygiene, perceived their oral health as good, and had high self-efficacy.

**Clinical relevance:**

This study contributes to increased knowledge about HNC patients' self-perceived oral health and ability to maintain good oral hygiene during cancer treatment. Such knowledge can be used in the development of customized oral care protocols, which in turn may have a positive impact on both oral health and quality of life.

## Introduction

Head and neck cancers (HNC) are malignant tumours located in areas such as the oral cavity, oropharynx, larynx, nasopharynx and salivary glands. These malignancies collectively rank as the sixth most common type of cancer globally, with more than 946.000 new cases annually, accounting for approximately 5% of all cancer cases and occurring in more than twice as many men as women (1). In Sweden, the annual incidence is more than 1,700 HNC cases, corresponding to 2.3% of all cancer cases nationally (2).

Treatment of HNC typically include combinations of radiotherapy (RT), surgery and chemotherapy (CT), and regimens vary depending on several factors such as tumor type, location and the patient's overall health status.

HNC treatment causes several acute adverse effects, including oral mucositis, a painful inflammation that ranges from erythema to severe ulcerations of the mucosal membranes in the mouth and throat. Oral mucositis compromises the protective mucosal barrier and facilitates colonization of pathogenic microorganisms originating from dental plaque, which may aggravate ulcerations and lead to systemic infections such as sepsis (3). Oral mucositis usually improves 4-6 weeks after completed HNC treatment (4). A recent meta-analysis reported a prevalence of oral mucositis of 87% in HNC patients (5).

Reduced salivary secretion, taste alterations or loss of taste, difficulties in chewing and swallowing, and limited ability to open the mouth are more long-lasting or persistent side-effects after HNC treatment (4, 6). In addition to these physical challenges, HNC and its treatment also causes psychological symptoms like distress, anxiety, and depression (7, 8), which can impact oral health behaviors (9).

At the time of diagnosis, patients with HNC often have substantial dental treatment needs (10, 11). Poor oral hygiene with high levels of plaque and gingival inflammation has been reported prior to HNC treatment (12, 13). In a previous study, 42% of patients with HNC reported brushing their teeth twice a day and performing daily interdental cleaning at the time of diagnosis (14).

In Sweden, pre-treatment dental assessment and care are considered standard of care for patients with HNC (2). To establish adequate oral hygiene, dentistry needs to inform about common side-effects of HNC treatment and provide individualized information and instructions regarding toothbrushing, interdental cleaning, moisturisation of the oral cavity, diet, and fluoride administration. Developing guidelines for maintaining oral hygiene, ensuring regular dental visits during HNC treatment, and providing preventive care and long-term follow-up have been proposed as important components for comprehensive HNC care (15, 16).

The concept of self-perceived oral health, encompassing oral health-related perceptions, attitudes, and behaviors, has been used in previous studies (17, 18). It reflects an individual's subjective assessment of their own oral health status. Positive oral health-related perceptions, attitudes and behaviours have been associated with more favourable oral hygiene habits and better oral health status (18). Individuals with poor oral hygiene and gingival conditions report less frequent toothbrushing and have an overall more negative attitude towards their oral health, compared with individuals who have low levels of plaque and gingivitis (18, 19). However, an individual's subjective belief about their oral health does not always correspond to clinical findings and assessments made by dental staff (20). Little is known about self-perceived oral health and oral health-related perceptions, attitudes, behaviors, and knowledge in patients with HNC.

Self-efficacy refers to an individual's perceived ability or confidence to manage different life events and to carry out the behavioral actions necessary to achieve specific outcomes or health goals (21). Individuals with high confidence in their own abilities strongly believe that they can manage demanding tasks and tend to view such challenges as problems to be mastered rather than threats to be avoided. In contrast, individuals with low confidence in their abilities may be more inclined to give up or fail to adapt their strategies when facing difficult challenges (21). General self-efficacy (GSE) reflects a broader, generalized sense of competence in contrast to the domain-specific self-efficacy described by Bandura (21). Both perspectives converge on the predictive role of self-efficacy in coping, motivation, and emotional well-being (22). To our knowledge, studies investigating self-efficacy in relation to HNC and its treatment are lacking.

The aim of this study was to explore self-perceived oral health and general self-efficacy and relate this to dental plaque, gingival inflammation and oral mucositis in head and neck cancer patients in Sweden, before, during, and three months after treatment.

## Material and methods

This observational study was embedded within a larger research project aiming at evaluating an oral care protocol for patients undergoing treatment for HNC. The study was conducted in accordance with the World Medical Association Declaration of Helsinki: Ethical principles for medical research involving human subjects, and was approved by the Regional Ethics Committee, University of Gothenburg (831–16).

Patients were consecutively recruited from five regions in Sweden (Jönköping, Kalmar, Lund, Stockholm, Umeå) prior to the initiation of cancer treatment. Inclusion criteria were age ≥ 18 years and scheduled to receive curative HNC cancer treatment including RT. Exclusion criteria were recurrent cancer and/or severe cognitive impairment (e.g dementia, brain injury, or difficulty reading and understanding written text). Eligible patients received both oral and written information about the study, and informed consent was obtained. Patients were recruited from April 2018 to May 2023. This approach was used to reduce the risk of selection bias, since all patients meeting the inclusion criteria during the study period were offered participation without subjective selection.

### Patient characteristics

At baseline, data were collected on HNC diagnosis, tumor site, radiation dose, surgery, chemotherapy, height, weight, smoking status (current, former, never), alcohol intake, comorbidities, and current medication. Medical conditions and medications for each patient were classified using the International Classification of Diseases (ICD) and the Anatomical Therapeutic Chemical (ATC) classification system. If patients were unable to provide information, their medical records were reviewed at the time of data collection. These data were used to describe the patient population.

### Head neck cancer treatment

All patients received either Intensity Modulated Radiation Therapy IMRT or Volumetric Modulated Arc Therapy (VMAT), at a dose rate of 2 Gy/day, five days per week for 5–7 weeks, totaling 50–68 Gy. Patients receiving chemotherapy were administered Cisplatin weekly during RT (CRT). Five patients underwent surgery followed by RT or CRT. One patient with oral cancer received mandibulectomy, another maxillectomy. One patient with nasopharyngeal cancer and one with oropharyngeal cancer underwent tumor resection, while one patient with salivary gland cancer received parotidectomy.

### Dental examination and treatment

Before initiating HNC treatment, all patients underwent a dental examination including radiographs. If necessary, periodontal treatment and extraction of teeth with poor or uncertain prognosis were performed to reduce the risk of oral infections (4). These pre-treatment examinations and procedures, as well as professional oral care during HNC treatment, were covered by the Swedish national health insurance and provided free of charge for the patient.

### Oral care protocol

Professional oral care was provided weekly during HNC treatment and included plaque removal, oral hygiene instructions, fluoride recommendations, and moisturizing of the oral cavity. Plaque and gingival inflammation were assessed prior to each professional oral care session, with at least one week between appointments.

### Clinical data collection

Clinical examinations were conducted at Specialist Dental Clinics for Orofacial Medicine within the same hospital where patients received HNC treatment. Data collection was performed by six dental hygienists at their respective clinic. Prior to study initiation, all dental hygienists received standardized training conducted by author CK, a dental hygienist with extensive experience in the oral care of head and neck cancer patients at a specialist clinic for orofacial medicine. Training included detailed instructions on scoring plaque, gingival inflammation, and oral mucositis, optimizing clinical workflows, and administering of the SPOH and GSE questionnaires. Training was delivered in person and followed up by a digital meeting at the start of recruitment. Digital workshops were held throughout the study to reinforce training and address questions. Calibration for oral mucositis scoring was conducted by reviewing photographs of varying severity of oral mucositis and discussing them in groups in collaboration with DDS. Richard Olofsson, specializing in orofacial medicine.

All oral/dental examinations were conducted with patients seated or reclined in a dental chair. Missed and filled teeth were recorded visually and using existing x-rays. Plaque and gingival inflammation were registered at baseline, week 6 of HNC treatment, and 3 months post-treatment on the six Ramfjord index teeth (23). Plaque was scored using the Plaque Index (PI), graded 0–3 based on Silness and Löe (24), on four surfaces of each tooth (labial/buccal, palatinal/lingual, mesial and distal). If a target tooth was missing, adjacent teeth were examined when possible. A plaque score of 0 indicated no plaque, score 1 indicated thin plaque layer detected by using a probe. Score 2 indicated moderate plaque visible to the naked eye and score 3 indicated an abundance of plaque. Gingival inflammation was scored based on Löe and Silness (25), on four surfaces of each tooth (labial/buccal, palatinal/lingual, mesial and distal). A score of 0 indicated no inflammation, score 1 indicated mild inflammation, score 2 indicated moderate inflammation with bleeding on probing, and score 3 severe inflammation. Mean scores were calculated by dividing the total score by the number of assessed surfaces.

Oral mucositis was assessed at the appointments for professional oral care at baseline, weekly during treatment, and one and three months after completed treatment using the Oral Mucositis Assessment Scale (OMAS) (26), where oral mucositis is registered on nine intraoral sites: (1) upper lip, (2) lower lip, (3) left side of the buccal mucosa, (4) right side of the buccal mucosa, (5) left ventral and dorsal side of the tongue, (6) right ventral and dorsal side of the tongue, (7) floor of the mouth, (8) soft palate and (9) hard palate. Ulceration was scored from 0 to 3, (0 = no ulceration, 1 = ulceration <1 cm^2^, 2 = ulceration 1–3 cm^2^, 3 = ulceration > 3 cm^2^), and erythema was scored from 0 to 2 (0 = no erythema, 1 = mild erythema, 2 = severe erythema) resulting in an ulceration score ranging from 0 to 27 and an erythema score ranging from 0 to 18. Mean scores for erythema and ulcerations were calculated separately at each time-point. Ulceration scores of ≥2 were considered indicative of severe oral mucositis.

### Patient reported outcome measures

The original version of the self- perceived oral health questionnaire (SPOH) consists of 27 items (17, 27). Thirteen items targeting young people were excluded by author AA and JSA, leaving 14 items. Each item had four fixed response options: two positively inclined and two negatively inclined. Five items addressed oral health-related perceptions, three attitudes, two oral health related behaviors (toothbrushing and interdental cleaning), and four items covered knowledge about caries and gum diseases, and knowledge regarding the effects of diet and beverages on dental health. Patients completed the questionnaire in connection with the visit to the dental hygienist at baseline, week 6 during treatment and 3 months post treatment.

To assess the patient's perceived ability to cope with life challenges, the General Self-Efficacy (GSE) scale was used. It consists of 10 items scored from 1 (totally disagree) to 4 (totally agree), yielding a total score between 10 and 40. Higher scores indicate greater self-efficacy. While no fixed threshold exists, scores above 29 are generally considered as high (28, 29). The GSE scale has been validated in Swedish (30). Patients completed the GSE at the same time points as the SPOH. Variations in patient numbers across time points were primarily due to health-related inability to attend appointments.

### Statistical analysis

Mean values and standard deviations (SD) were calculated for number of teeth, missed and filled teeth, plaque, gingival inflammation, and oral mucositis (erythema and ulceration). Differences in plaque and gingival inflammation across time points (baseline, week 6 during treatment and 3 months post treatment) were analyzed using the Friedman Test with the Wilcoxon signed rank test as a *post-hoc* analysis. SPOH responses were trichotomized: the two positively inclined response options were analyzed separately. While the two negatively inclined options were merged due to low response rates. Changes in answers to SPOH items between time-points were analyzed using McNemar-Bowker's test. Differences in GSE scores between time point were analysed using Friedman test. Differences in mean scores between 3 months post-treatment and week 6 during treatment were calculated for plaque, gingival inflammation and oral mucositis. One-way ANOVA with Bonferroni correction was used to analyze differences in these clinical variables and the answers to the fourteen SPOH items at week 6. Pearsońs correlation was used to assess associations between clinical variables and GSE score at week 6. A significance level of *p* < 0.05 was applied to all tests.

## Results

Seventy-five patients were included: 57 men and 18 women, with mean age of 59 years (range 22–79 years). The majority were diagnosed with oropharynx cancer (*n* = 54) or oral cancer (*n* = 7). Chemoradiotherapy was the most common treatment modality (*n* = 42) (Table 1).

**Table 1:** Sex, tumor location, treatment, diseases/conditions, medicines, smoking, and alcohol use for the 75 patients with head and neck cancer.

Variable	Patient descriptives	*n* (%)
Sex	Men/Women	57/18 (76/24)
Tumor location	Oropharynx	54 (72)
	Oral	7 (9)
	Larynx	7 (9)
	Salivary glands	3 (4)
	HNCUP^a^	3 (4)
	Nasopharynx	1 (2)
Treatment	Chemotherapy + Radiotherapy	42 (56)
	Radiotherapy	27 (36)
	Radiotherapy + Surgery	4 (5.3)
	Radiotherapy + Brachytherapy	1 (1.3)
	Radiotherapy + Chemotherapy + Surgery	1 (1.3)
Medical conditions	No comorbidity	45 (60)
	1 comorbidity	10 (13)
	2–4 comorbidities	20 (27)
Current medication	No medicines	20 (27)
	1–3 medicines	24 (32)
	4–8 medicines	31 (41)
Smoking	Never	44 (59)
	Former	28 (37)
	Current	3 (4)
Alcohol use	None	28 (37)
	Liquor	4 (5)
	Other (wine, beer)	34 (45)
	Liquor + other	9 (12)

a HNCUP, head and neck carcinoma of unknown primary.

At baseline, 45 patients (60%) had no comorbidities (Table 1). Hypertension was the most frequent condition (*n* = 13, 16%), followed by cardiovascular disease (*n* = 6, 7%), type 2 diabetes mellitus (*n* = 6, 7%), and asthma (*n* = 4, 5%). The most common medications were antihypertensives (*n* = 19, 24%), analgesics (*n* = 12, 15%), and statins (*n* = 6, 8%). Forty-four patients (59%) were never smokers, 28 (37%) were former smokers of whom 5 (15%) had quit smoking within the past 3 months, and 3 patients (4%) were current smokers.

### Clinical oral status

At baseline, the mean ± SD number of teeth was 27 ± 4 (median 27, range 16–32), and the mean number of filled teeth was 13 ± 7 (median 13, range 0–28). Plaque and gingival inflammation scores were low at all time-points, with the lowest mean scores observed 3 months post-treatment (Table 2). Plaque scores were significantly lower at 3 months post treatment compared to baseline (*p* < 0.01) and week 6 during treatment (*p* < 0.01). Gingival inflammation scores were also significantly lower at 3 months post treatment compared with baseline (*p* < 0.01).

**Table 2:** Dental plaque and gingival inflammation at baseline (BL), week 6 during treatment and 3 months post treatment for the 75 patients.

Variable	Baseline	*p*-value	Week 6	*p*-value	3 months post	*p*-value
		BL-W6		W6–3 mo		BL-3 mo
Dental plaque	(*n* = 74)		(*n* = 61)		(*n* = 71)	
Mean ± SD	0.45 ± 0.55	N.S	0.40 ± 0.39	*p* < 0.01	0.29 ± 0.35	*p* < 0.01
Median	0.25		0.29		0.17	
Range	0.00–2.50		0.00–1.63		0.00–1.88	
Gingival inflammation	(*n* = 72)		(*n* = 60)		(*n* = 71)	
Mean ± SD	0.36 ± 0.47	N.S	0.24 ± 0.30	N.S	0.22 ± 0.30	*p* < 0.01
Median	0.19		0.15		0.08	
Range	0.00–2.00		0.00–1.33		0.00–1.50	

Differences in plaque and gingival inflammation between time-points (baseline, week 6 during treatment and 3 months post treatment) were analysed using the Friedman Test with Wilcoxon signed rank test as a *post-hoc* test.

### Oral mucositis

**Figure 1:**
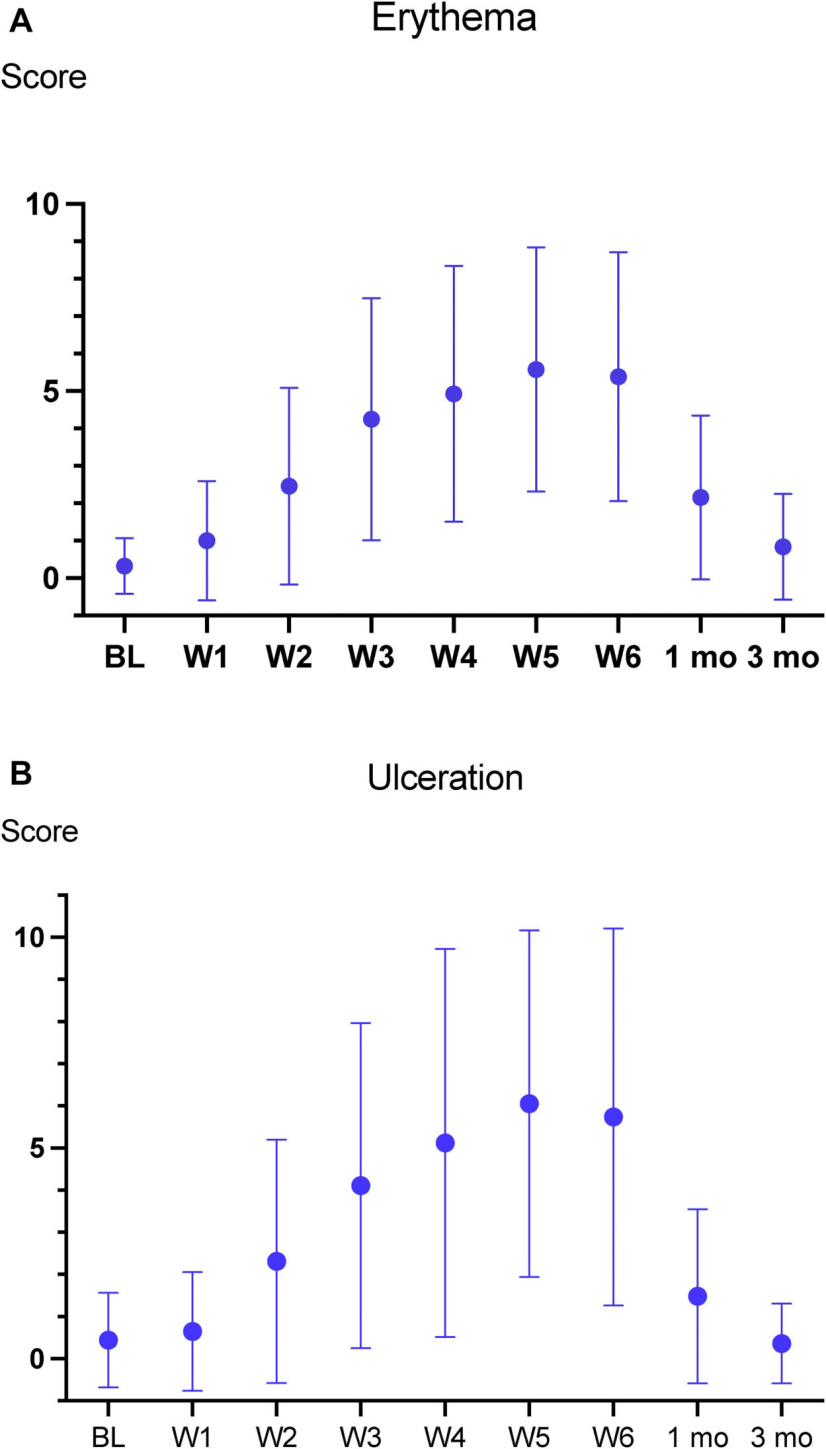
**(A)** Erythema scores (mean ± SD) at baseline (BL), during oncological treatment (W1–W6), and one- and three-months post treatment. **(B)** Ulcerations scores (Mean ± SD) at baseline (BL), during oncological treatment (W1–W6), and one- and three-months post treatment.

Mean erythema and ulceration scores increased progressively during treatment (Figure 1A). Erythema scores ≥2 were present in 35 patients (49%) at week 2, rising to 55 patients (75%) at week 3. The largest increase occurred between weeks 2 and 3, with scores peaking at week 5, when 61 patients (92%) exhibited severe erythema (≥2). One month post-treatment, erythema scores had decreased (Figure 1A). Ulceration followed a similar pattern: 35 patients (49%) had scores ≥2 at week 2, peaking at week 5 with 54 patients (82%) (Figure 1B). As for erythema, the ulceration scores had decreased one month after treatment. The variation in scores for both erythema and ulceration was substantial.

### **Perceptions** (SPOH questionnaire, question 1–5)

A significantly lower proportion of patients rated their oral health as good/fairly good at week 6 compared with baseline (74% vs. 91%*p* < 0.05) (Table 3a). Three months post- treatment, this proportion increased compared with week 6 (87% vs. 74% *p* < 0.01) and was comparable to baseline (91%). More than 70% were satisfied with the esthetics of their teeth at all time-points. Approximately 90% consistently reported high or fairly high perceived control over their oral health. The proportion reporting taking good care of their teeth was high at baseline and had increased 3 months post treatment (*p* < 0.01).

**Table 3a:** Shows differences in SPOH questionnaire response for the 75 HNC patients. Question 1–5 = Perceptions, Question 6–8 = Attitudes. Responses were collected at baseline, at week 6 during treatment and 3 months after completed treatment.

Question no	SPOH question	Baseline	Week 6	3 months	Significant differences
		*n* (%)	*n* (%)	*n* (%)	
1	How do you consider your oral health?				
	Good	31 (43)	11 (18)	23 (33)	BL-Week 6*
	Fairly good	35 (48)	34 (56)	37 (54)	Week 6–3 mo**
	Quite poor/poor	7 (9)	16 (26)	9 (13)	
2	Are you satisfied with the esthetics of your teeth?				
	Very satisfied	14 (19)	15 (25)	15 (22)	N.S
	Fairly satisfied	47 (65)	31 (50)	42 (61)	
	Quite/very dissatisfied	12 (16)	15 (25)	12 (17)	
3	How often do your gums bleed when you brush?				
	Seldom/never	39 (54)	30 (49)	34 (50)	N.S
	Once a month	20 (27)	13 (21)	23 (33)	
	Every day/few times a week	14 (19)	18 (30)	12 (17)	
4	How do you consider your possibility to impact on your oral health?				
	High	37 (51)	34 (56)	41 (59)	N.S
	Fairly high	33 (46)	23 (38)	26 (38)	
	Quite low/low	2 (3)	4 (6)	2 (3)	
5	How well do you think you take care of your teeth?				
	Well	32 (44)	29 (47)	41 (59)	BL- 3 mo**
	Fairly well	37 (51)	26 (43)	26 (38)	
	Quite bad/bad	4 (5)	6 (10)	2 (3)	
6	How important is it for you to have healthy oral conditions?				
	Very important	56 (77)	48 (79)	54 (78)	N.S
	Fairly important	17 (23)	13 (21)	15 (22)	
	Less important/not at all	0 (0)	0 (0)	0 (0)	
7	How important is it for you to clean your teeth?				
	Very important	47 (64)	45 (74)	48 (70)	N.S
	Fairly important	24 (33)	13 (21)	20 (29)	
	Less important/not at all	2 (3)	3 (5)	1 (1)	
8	It is important to me to have sound teeth:				
	Correspond precisely	62 (85)	55 (90)	61 (88)	N.S
	Correspond roughly	11 (15)	6 (10)	8 (12)	
	Correspond poorly/not at all	0 (0)	0 (0)	0 (0)	

McNemar's test was used to access statistically significant differences between baseline and week 6, baseline and 3 months, and week 6 and 3 months shown by the *p*-values.

* *p* < 0.05.

** *p* < 0.01.

*** *p* < 0.001.

### Attitudes **(**question 6–8 in the SPOH questionnaire)

At baseline, all patients rated healthy oral conditions as very or fairly important, and 97% considered cleaning their teeth very or fairly important (Table 3a). All patients responded precisely or roughly to the statement about importance for them to have sound teeth. No statistically significant differences were observed across time-points.

### Behaviors (question 9–10 in the SPOH questionnaire)

At baseline, 95% reported brushing their teeth at least twice a day with no statistically significant changes across timepoints (Table 3b). The proportion reporting daily interdental cleaning had increased at week 6 during treatment compared with baseline (72% vs. 51%, *p* < 0.05), and remained elevated 3 months post treatment (*p* < 0.01).

**Table 3b:** Shows differences in SPOH questionnaire response for the HNC 75 patients. Question 9–10 = Behavior, Question 11–14 = Knowledge. Responses were collected at baseline, at week 6 during treatment and 3 months after completed treatment.

Question no	SPOH question	Baseline	Week 6	3 months	Significant differences
		*n* (%)	*n* (%)	*n* (%)	
9	How often do you brush your teeth?				
	2 times/day or more	69 (95)	58 (95)	67 (98)	N.S
	Once a day	3 (4)	2 (3)	1 (1)	
	Few days a week/or more Seldom	1 (1)	1 (2)	1 (1)	
10	How often do you clean (interdentally) between your teeth:				
	Every day	37 (51)	44 (72)	46 (67)	BL-Week 6*
	At least once a week	25 (34)	13 (21)	20 (29)	BL- 3 mo**
	A few times a month/or more seldom	11 (15)	4 (7)	3 (4)	
11	My knowledge about gum diseases is:				
	Good	30 (41)	13 (21)	14 (20)	BL-Week 6**
					BL-3 mo**
	Fairly good	34 (47)	29 (48)	38 (55)	
	Quite poor/poor	9 (12)	19 (31)	17 (25)	
12	My knowledge about caries is:				
	Good	11 (15)	21 (34)	32 (46)	BL-Week 6***
					BL- 3 mo***
	Fairly good	30 (41)	31 (51)	27 (39)	
	Quite poor/poor	32 (44)	9 (15)	10 (15)	Week 6–3 mo*
13	My knowledge about how my dietary habits affect my teeth is:				
	Good	19 (26)	22 (36)	33 (48)	BL-Week 6*
					BL-3 mo***
	Fairly good	36 (49)	32 (53)	28 (41)	
	Quite poor/poor	18 (25)	7 (11)	8 (11)	
14	My knowledge about what I am drinking affect my teeth is:				
	Good	33 (45)	25 (41)	37 (54)	N.S
	Fairly good	32 (44)	31 (51)	26 (38)	
	Quite poor/poor	8 (11)	5 (8)	6 (8)	

McNemar's test was used to access statistically significant differences between baseline and week 6, baseline and 3 months, and week 6 and 3 months shown by the *p*-values.

* *p* < 0.05.

** *p* < 0.01.

*** *p* < 0.001.

### Knowledge (question 11–14 in the SPOH questionnaire)

At baseline, 88% considered their knowledge of gum disease good or fairly good, but proportions declined at week 6 (*p* < 0.01) and 3 months post- treatment (*p* < 0.05) (Table 3b). In contrast, the patients considered that their knowledge about caries had increased significantly at week 6 (*p* < 0.001) and remained higher at 3 months (*p* < 0.001). Knowledge of dietary habits and how it affects the teeth was rated fairly good or good at baseline, increasing significantly at week 6 (*p* < 0.05) and 3 months post treatment (*p* < 0.001). Knowledge about what they drink and how it can affect the teeth was rated good or fairly good by 89% at baseline, with no statistically significant changes across time points.

### Self-perceived oral health and clinical variables

No statistically or clinically significant differences were found between changes in plaque, gingival inflammation or oral mucositis (week 6 vs. 3 months post-treatment) and oral health-related perceptions, attitudes, behaviors and knowledge (ANOVA with Bonferroni correction).

### General self-efficacy GSE

At baseline, the mean GSE score was 34 ± 5, indicating high self-efficacy, with minimal changes across time points. A significant negative correlation was observed between GSE score at week 6 and changes in gingival inflammation from week 6–3 months post-treatment (Pearson's *r* = –0.315, *p* < 0.05).

## Discussion

To the best of our knowledge, this is the first study to explore oral hygiene levels, self-efficacy and oral health-related perceptions, attitudes, behaviors, and knowledge among patients with HNC. At baseline, most patients had high self-efficacy and perceived their oral health as good, alongside positive oral health-related attitudes, perceptions, and behaviors. They also perceived their knowledge of caries, gum diseases, and dietary habits to be fairly good or good. Only minor changes were observed over time, despite the peak of debilitating side effects at week 5–6 of treatment, when 82% of the patients presented with ulceration scores of ≥2. Symptoms associated with the peak of oral mucositis may include severe oral pain, difficulty opening the mouth, and fatigue, all of which can negatively interfere with daily oral care routines. It is therefore reasonable to expect a reduction in oral hygiene behaviours at this time point during treatment.

Contrary to these expectations, behaviors such as toothbrushing and interdental cleaning were largely maintained during treatment despite the severe side effects. This suggests that positive attitudes and sufficient knowledge may help patients overcome physical barriers to oral care. The SPOH questionnaire captures the individual's perceptions of their oral health and can be integrated into preventive and oral health-promotive strategies. While instruments such as the Oral Health Impact Profile (OHIP) (31) and Oral Impacts on Daily Performances (OIDP) (32) are widely used, they measure oral health-related quality of life (OHRQoL) rather than perceptions, attitudes and behaviors towards oral health. Although SPOH dimensions -perceived oral health, attitudes, behaviors, and knowledge- were analyzed separately, they are closely interrelated and often influence one another. For example, knowledge about oral health may shape attitudes, which in turn affect behaviors and ultimately perception of oral health.

The mean plaque and gingival inflammation scores at baseline were low and notably lower than those reported in a recent Chinese study on HNC patients (33). Several factors may explain this difference. In our study, patients underwent a dental examination and received necessary treatment before initiating HNC treatment, including education on maintaining good oral hygiene, provided free of charge by dental hygienists at Specialist Dental Clinics for Orofacial medicine. In contrast, in the study by Jiang et al. (33), patients received oral hygiene instructions by dental hygienist students and were advised to seek pre-dental treatment care at their own expense. Whether the patient followed this recommendation or not was not stated in their study. Furthermore, the high rate of routine dental attendance among Swedish adults (34), supported by a public dental care system, likely contributes to better oral health outcomes in the present study. In China, there is limited access to affordable dental care and oral health is generally poorer (35).

In the present study, the patients visited the dental hygienist weekly during HNC treatment for professional oral care, and when needed, reinforcement of oral hygiene instructions. Previous research has shown that weekly professional oral care during the HNC treatment, results in lower plaque levels compared to patients who only receive instructions prior to treatment (36). Both our finding and those of Sohn et al. (36), supports the effectiveness of weekly professional care in maintaining low levels of plaque and gingival inflammation, underscoring the importance of including dental professionals in the multidisciplinary HNC care team, which has been suggested also in other studies (37, 38).

Although higher levels of plaque and gingival inflammation were expected at week 6 due to oral mucositis-related discomfort, this was not observed. One possible explanation is that patients, concerned about exacerbating oral symptoms or infection adhered strictly to oral hygiene routines. In contrast, a Chinese study reported visible plaque in 66% of patients at week 6 (39). Differences in access to professional care may account for this discrepancy: in our study, weekly free-of-charge visits provided patients with strategies to maintain good oral hygiene despite adverse conditions, whereas in the study by Liu et al. (39), oral care was limited to wiping the teeth with water-soaked cotton buds.

Poor oral hygiene can exacerbate oral mucositis; however, even with low levels of plaque and gingival inflammation (15, 40) oral mucositis with ulceration scores of ≥2 was prevalent in our study. The primary cause of oral mucositis is the cytotoxic effect of radiotherapy and/or chemotherapy on rapidly dividing epithelial cells of the oral mucosa (3). The radiotherapy also causes damage to salivary glands resulting in reduced salivary secretion, reduced pH and buffer capacity, an altered electrolyte composition (41, 42),, and disrupted mucin networks (41), impairing protective functions of saliva facilitating colonization by pathogenic microorganisms such as Gram negative, anaerobic bacteria (43).

Our findings indicate that HNC patients maintained positive oral health-related perceptions, attitudes and behaviours, and low levels of plaque and gingival inflammation, which aligns with previous studies in non-HNC populations (18). Variability in SPOH responses was minimal across time points, except for perceived oral health, which declined significant from 90% at baseline to 74% week 6. Interestingly, a higher proportion of HNC patients considered healthy oral conditions important (77%–79%) compared to individuals without HNC (71%) (18). Due to the low variability in SPOH responses and generally low levels of plaque and gingival inflammation we decided to analyse differences between responses to SPOH items at week 6 and differences in plaque, gingival inflammation between week 6 during treatment and 3 months post treatment. During that time, patients visited the dental hygienist only a few times rather than weekly as they did during treatment. This reduced frequency may have led patients to revert to their previous oral hygiene habits. However, no statistically significant differences were observed between SPOH responses and differences in clinical variables. A plausible explanation is that the SPOH questionnaire was designed for general populations and may not have been sufficiently sensitive to detect changes in oral health perceptions among HNC patients during treatment. Social desirability bias may also have influenced responses: patients receiving repeated oral hygiene instructions and professional care may have been aware of the expected answers and thus reported more favourable attitudes and behaviours (44).

Most of the HNC patients reported high self-efficacy, with a mean score of 34 at baseline, consistent with a previous HNC study (45) and slightly higher than the Norwegian population mean of 29 (46). High self-efficacy may have contributed to confidence in managing treatment and determination to maintain oral hygiene despite pain and discomfort. However, HNC survivors often experience persistent oral health issues post-treatment, such as xerostomia and dental caries. The absence of clear post-treatment guidelines and continued dental care subsidies creates uncertainty regarding long-term oral health, highlighting the need for individualized and affordable care strategies.

### Strengths and limitations

This multicenter study recruited patients from 5 diverse geographical locations in Sweden, enhancing external validity and enabling inclusion of a larger sample. Another strength is the combined assessment of self-perceived oral health and clinical outcomes, providing a comprehensive understanding of oral health in HNC patients. No priori sample size calculation was performed, instead all eligible patients during the study period were included. Given the limited prior data in this area, the results should be interpreted cautiously. A potential limitation is self-selection bias (47), as participants may have been healthier and more motivated than non-participants, reducing representativeness. Additionally, weekly professional oral care may have minimized differences in levels of plaque and gingival inflammation, a HNC control group would have been preferable for comparison but withholding care (36–38) would have been unethical. Clinical calibration was performed for oral mucositis scoring, however, inter-rater reliability was not assessed, which may affect reproducibility. Calibration for registration of plaque and gingival inflammation was not conducted, as these assessments were considered routine for dental hygienists.

### Clinical relevance

This study provides valuable insights into HNC patients' ability to maintain good oral hygiene and their self-perceived oral health during cancer treatment. These findings can be used in the development of tailored oral care protocols, potentially improving both oral health and quality of life.

## Conclusion

Despite significant treatment-related challenges, HNC patients' demonstrated high self-efficacy and positive oral health perceptions, reflected in favorable clinical outcomes, even during severe oral mucositis. The limited variability in both clinical and self-reported measures suggests a resilient and motivated patient population. Asking the patients about their perceived oral health and their confidence in managing challenges and stressful situations can support patient-centred oral care strategies during HNC treatment.

## Data Availability

The raw data supporting the conclusions of this article will be made available by the authors, without undue reservation.
