# Supplementary material for: Self-perceived oral health, general self-efficacy, and their relations to oral health conditions in head and neck cancer patients in Sweden—a prospective observational study
Source: Front Oral Health. 2026 Jan 5;6:1693673. doi: 10.3389/froh.2025.1693673 (PMC12813088; doi:10.3389/froh.2025.1693673)
Supplement: Supplementary file 2 [file Datasheet2.pdf]

Self-perceived oral health (SPOH)

Please mark only <u>one</u> alternative

Questions about your perceived oral health

1. How do you consider your oral health?
   - ☐ Good
   - ☐ Fairly good
   - ☐ Quite poor
   - ☐ Poor

2. Are you satisfied with the esthetics of your teeth?
   - ☐ Very satisfied
   - ☐ Fairly satisfied
   - ☐ Quite dissatisfied
   - ☐ Very dissatisfied

3. How often do your gums bleed when you brush?
   - ☐ Every day
   - ☐ Few times a week
   - ☐ Once a month
   - ☐ Seldom/Never

4. How do you consider your possibility to impact on your oral health?
   - ☐ High
   - ☐ Fairly high
   - ☐ Quite low
   - ☐ Low

5. How well do you think you take care of your teeth?
   - ☐ Well
   - ☐ Fairly well
   - ☐ Quite bad
   - ☐ Bad

Questions about your assessment of how you take care of your mouth

6. How important is it for you to have healthy oral conditions?
   - ☐ Very important
   - ☐ Fairly important
   - ☐ Less important
   - ☐ Not at all important

7. How important is it for you to clean your teeth?
   - ☐ Very important
   - ☐ Fairly important
   - ☐ Less important
   - ☐ Not at all important

Modified from Östberg AL. On self-perceived oral health in Swedish adolescents. Swed Dent J Suppl. 2002; 155: 1-87

8. It is important for me to have sound teeth.
☐ Correspond precisely
☐ Correspond roughly
☐ Correspond poorly
☐ Correspond not at all

9. How often do you brush your teeth?
☐ 2 times/day or more often
☐ Once a day
☐ Few days a week
☐ Once a week or more seldom

10. How often do you clean (interdentally) between the teeth?
☐ Every day
☐ At least once a week
☐ A few times a month
☐ Seldom/never

Your assessment of your knowledge
11. My knowledge about gum diseases is:
☐ Good
☐ Fairly good
☐ Quite poor
☐ Poor

12. My knowledge about caries is:
☐ Good
☐ Fairly good
☐ Quite poor
☐ Poor

13. My knowledge about how my dietary habits affect my teeth is:
☐ Good
☐ Fairly good
☐ Quite poor
☐ Poor

14. My knowledge about what I am drinking affect my teeth is:
☐ Good
☐ Fairly good
☐ Quite poor
☐ Poor

Modified from Östberg AL. On self-perceived oral health in Swedish adolescents. Swed Dent J Suppl. 2002; 155: 1-87
